# mRNA Expression Levels in Failing Human Hearts Predict Cellular Electrophysiological Remodeling: A Population-Based Simulation Study

**DOI:** 10.1371/journal.pone.0056359

**Published:** 2013-02-20

**Authors:** John Walmsley, Jose F. Rodriguez, Gary R. Mirams, Kevin Burrage, Igor R. Efimov, Blanca Rodriguez

**Affiliations:** 1 Department of Computer Science, University of Oxford, Oxford, United Kingdom; 2 Aragón Institute of Engineering Research, University of Zaragoza, Zaragoza, Spain; 3 Biomedical Research Networking Center in Bioengineering, Biomaterials and Nanomedicine, Zaragoza, Spain; 4 School of Mathematical Sciences, Queensland University of Technology, Brisbane, Queensland, Australia; 5 Department of Biomedical Engineering, Washington University in St. Louis, St. Louis, Missouri, United States of America; Baylor College of Medicine, United States of America

## Abstract

Differences in mRNA expression levels have been observed in failing versus non-failing human hearts for several membrane channel proteins and accessory subunits. These differences may play a causal role in electrophysiological changes observed in human heart failure and atrial fibrillation, such as action potential (AP) prolongation, increased AP triangulation, decreased intracellular calcium transient (CaT) magnitude and decreased CaT triangulation. Our goal is to investigate whether the information contained in mRNA measurements can be used to predict cardiac electrophysiological remodeling in heart failure using computational modeling. Using mRNA data recently obtained from failing and non-failing human hearts, we construct failing and non-failing cell populations incorporating natural variability and up/down regulation of channel conductivities. Six biomarkers are calculated for each cell in each population, at cycle lengths between 1500 ms and 300 ms. Regression analysis is performed to determine which ion channels drive biomarker variability in failing versus non-failing cardiomyocytes. Our models suggest that reported mRNA expression changes are consistent with AP prolongation, increased AP triangulation, increased CaT duration, decreased CaT triangulation and amplitude, and increased delay between AP and CaT upstrokes in the failing population. Regression analysis reveals that changes in AP biomarkers are driven primarily by reduction in I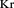, and changes in CaT biomarkers are driven predominantly by reduction in I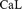 and SERCA. In particular, the role of I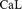 is pacing rate dependent. Additionally, alternans developed at fast pacing rates for both failing and non-failing cardiomyocytes, but the underlying mechanisms are different in control and heart failure.

## Introduction

A growing number of studies examine cardiac tissue mRNA expression levels in an attempt to characterize electrophysiological remodeling associated with disease conditions such as heart failure and atrial fibrillation [1]–[3]. Recent studies have shown alterations in expression of mRNA transcripts in human failing hearts compared to non-failing hearts [2], [3]. Furthermore, recent optical mapping measurements have also demonstrated differences in action potential (AP) and calcium transients (CaT) between failing and non-failing human hearts [4]–[6], with potentially pro-arrhythmic implications [7]–[10].

Linking mRNA changes to AP and CaT alterations has several complications, as recently reviewed by Nattel *et al* [11]. Firstly, mRNA expression is only one determinant factor of ion channel expression at the cell membrane. Translation rate, binding with channel subunits, methylation, membrane localization, trafficking and phosphorylation all affect the activity of ion channels. Secondly, expression of mRNA in cardiac cells has been reported to vary with circadian rhythm [12], [13]. And finally, mRNA expression levels might exhibit variability between tissue samples due to, for example, gender differences [1], [3], [14] and spatial location within the heart [2].

Despite these challenges, mRNA expression data provides information on heterogeneity and variability in the heart, in both healthy and diseased states. In this study, we aim to show how computational modelling can be used to understand the electrophysiological consequences of differences in mRNA expression levels between failing and non-failing human hearts. The methodology proposed is flexible and can be applied to any set of mRNA measurements and models. We illustrate its potential by focussing on the ventricular component of the dataset recently obtained by Ambrosi *et al* [3]. We will seek to establish whether information contained in mRNA transcription data can predict the functional differences in AP and CaT reported between failing and non-failing human hearts. We develop an approach that takes into account potential sources of variability and uncertainty in both change in conductance due to mRNA expression and electrophysiological measurements. We construct two cell model populations based on a human ventricular model [15]. Each population consists of an ensemble of cell models sharing the same equations representing ionic current kinetics but different conductance values. As in [16]–[20], we incorporate variability, which may arise from a variety of sources, by using a range of values for ion channel conductances rather than a single parameter value. The failing population is then constructed by additionally incorporating the upregulation or downregulation of conductances corresponding to mRNA expression changes reported in heart failure [3].

AP and CaT properties are quantified across both populations and compared to experimental measurements obtained in failing and non-failing human ventricles. We then predict the electrophysiological response of human ventricular myocytes at different stimulation rates and quantify the relative importance of each ionic current in determining AP and CaT differences in the human non-failing and failing cell populations.

## Methods

### mRNA Expression Data

mRNA expression data [3] are used to determine directions of changes in ionic conductances in failing versus non-failing human cardiomyocytes, as summarized in Table 1. Ambrosi *et al* [3] used a one-way analysis of variance followed by Tukey-Kramers test with a significance level of p 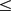 5.6
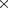
10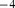 based on the Bonferroni correction 0.05. Three gene transcripts show a trend towards downregulation in heart failure, contributing to the conductances of L-type Ca current I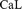 (G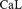), transient outward current I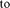 (G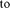), and the SERCA pump (J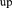). Gene transcripts for the rapid component of the delayed rectifier K current I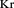 (HERG) also show a trend towards downregulation and are included in the simulations by altering the conductance of I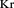 (G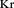). One gene transcript shows a trend towards upregulation in the heart failure cases, contributing to the conductance of the Na/Ca exchange current I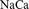 (G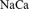). Two further currents are included in the analysis as they are active during the repolarisation phase of the action potential, the inward rectifier current I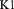 and the slow component of the delayed rectifier K current I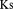. As gene transcripts associated with these channels show no trend in the data published in [3] they are considered to have natural variability only in both populations.

**Table 1:** Parameters under investigation.

Gene	Current	Parameter	Regulation in HF [3]
*KChIP2*	I_to_, I_CaL_ (a)	G_to_, G_CaL_	↓
*NCX1*	I_NaCa_	G_NaCa_	↑ (b)
*Serca2A*	SERCA	J_up_	↓
*Kv4.3*	I_to_	G_to_	↓ (c)
*Kv11.1*/*HERG*	*I*_Kr_	*G*_Kr_	↓ *(c)*
*Kv7.1*	I_Ks_	G_Ks_	–
*Kir2.1*	I_K1_	G_K1_	–

a) *KChIP2* has recently been shown to form an accessory subunit of I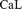 [40].

b) *NCX1* was downregulated in non-ischæmic cardiac myopathy patients but showed no difference from the non-failing group in ischæmic cardiac myopathy.

c) *Kv4.3* and *HERG* tended to be downregulated relative to the non-failing group however the difference was not statistically significant.

### Populations of Human Failing and Non-failing Cell Models

The O’Hara-Rudy (ORd) human ventricular endocardial model [15] is used to generate populations of non-failing and failing cell models. Each population contained 16384 models sharing the same equations as the ORd model but different parameter sets. The non-failing population is constructed by Monte-Carlo sampling of the parameters under investigation (G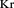, G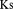, G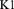, G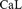, J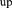, G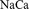, G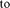) from a uniform distribution between 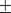
30% of the original parameter values, in line with previous studies [16]. This percent variation results in significant changes in biomarker properties across the population. The uniform distribution is used as we do not have prior information on the distribution of conductances in human cardiac myocytes. In the failing cell population, upregulation is sampled from a uniform distribution ranging from 0 to +60% change from the original parameter values and downregulation is sampled in the range -60% to 0% change from the original parameter values, in line with mean changes in mRNA expression data [3]. In the failing population, G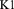 and G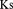 are sampled from the same distribution as in the non-failing population.

### Stimulation Protocol

The stimulation protocol mimics the one used by Lou *et al* [5] to obtain optical mapping measurements in failing versus non-failing cardiomyocytes. Cell models are stimulated for 100 paces at each of the following basic cycle lengths (BCLs) in a step protocol; 1500 ms, 1000 ms, 900 ms, 800 ms, 700 ms, 600 ms, 500 ms, 450 ms, 400 ms, 350 ms and 300 ms. A stimulus current of magnitude −80 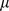
A/cm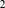 and duration 0.5 ms is applied at each pace.

### Biomarkers

**Figure 1:**
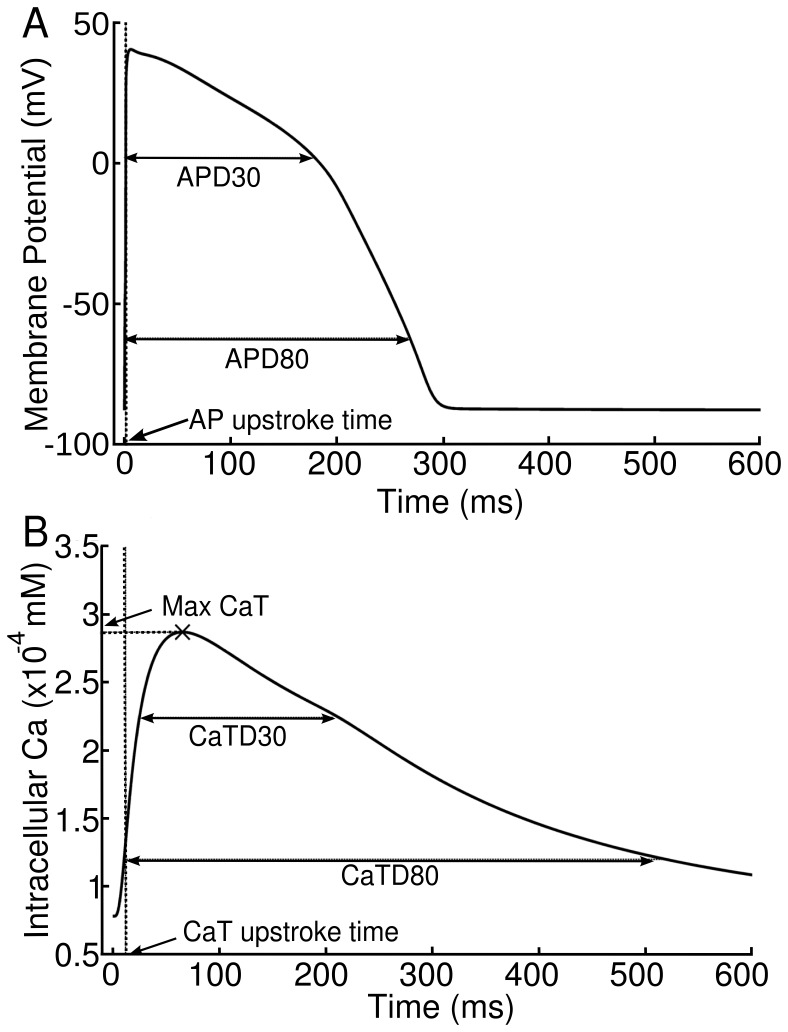
Biomarkers calculated from AP and CaT traces. A) shows APD80, APD30 and APD3080, and B) shows CaTD30, CaTD80, CaTD3080 and CaTMax. AP-CaT delay is the difference between the AP and CaT upstroke times.

As illustrated in Fig. 1, the following biomarkers are calculated from the two last action potentials simulated for each BCL: AP duration to 80% repolarisation (APD80), action potential triangulation (APD3080) defined as the ratio between AP duration to 30% repolarisation (APD30) and APD80, CaT duration to 80% of repolarisation (CaTD80), CaT triangulation (CaTD3080) defined as the ratio between CaT duration to 30% repolarisation (CaTD30) and CaTD80, maximum cytosolic calcium concentration (CaTMax) and the delay between the AP and CaT upstrokes (DAPCaT). The occurrence of voltage and calcium alternans is defined as a difference of 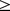
5 ms between the APD80 or CaTD80, respectively, in the penultimate and final APs.

### Regression Analysis

To quantify the relative importance of ionic conductances in determining changes in the biomarkers, linear regression [21], [22] is performed on the failing and non-failing populations, leaving out the cases showing alternans in APD80 or CaTD80. For each population, the following procedure is applied for each biomarker. Biomarker outputs are mean centred and normalised with respect to their standard deviation at each BCL, as in previous studies [21]. We seek linear regression coefficients 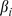 for each of the 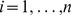 parameters under investigation such that for each of our 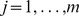 cells we have that the cell's predicted biomarker output 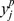 is given by
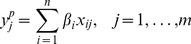
where 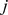 denotes the current cell, and 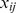 denotes the scaling of the 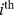 conductance for the 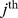 cell. This may be re-written as the matrix system



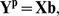
where the coefficients of 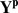 are 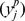, the coefficients of 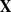 are 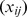 and the coefficients of 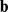 are 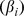. Let 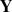 have coefficients 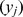, the computed biomarker values for each cell. We wish to find a solution 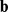 such that the least squares difference between 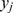 and 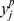 is minimised over all cells 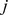. This solution is given by 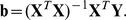 The vector 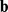 then contains our linear regression coefficients. This is repeated for each biomarker at each BCL.

To determine the effectiveness of this fit, the correlation coefficient 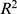 (R-squared) is computed for each linear regression fit. This coefficient is computed as
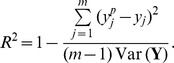



The closer 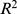 is to one, the more of the variance in the data is accounted for by the linear fit.

### Numerical Methods

All numerical simulations are performed using our open source cardiac simulation software Chaste [23]. A CellML [24] implementation of the ORd model is used from the functional curation database available within Chaste [25]. The ordinary differential equations are integrated using the numerical ODE software package CVODE (https://computation.llnl.gov/casc/sundials/) called from Chaste. A backward differentiation formula method is used to integrate the differential equations with absolute and relative error tolerances 10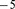 and 10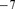 respectively. The output time step for biomarker calculation was 1 ms. All postprocessing of traces to calculate biomarker values was performed within the Chaste environment. Analysis of biomarkers was performed using Matlab. A Chaste user project containing the simulation codes used in this study is available to download from the Chaste website (http://www.cs.ox.ac.uk/chaste). Matlab data analysis scripts are also included. All simulations are performed on a 512 core cluster utilising 2.8GHz Intel processors at the Oxford Supercomputing Centre (OSC).

## Results

### Voltage and Calcium Transients in Non-failing and Failing Populations

**Figure 2:**
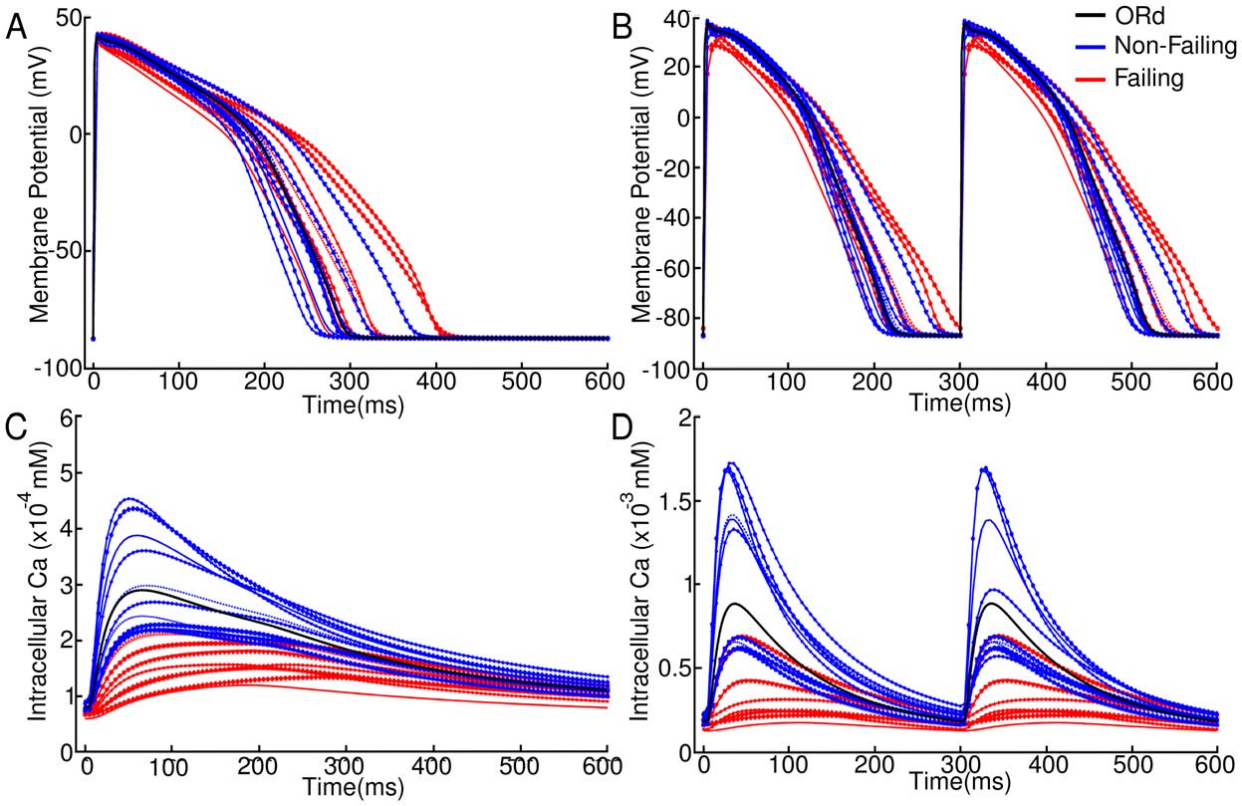
Sample AP and CaT traces obtained from the non-failing (blue) and failing (red) populations. Traces are shown for BCL = 1500 (A,C) and 300 ms (B,D). The AP for the standard ORd endocardial model is shown in black.

**Figure 3:**
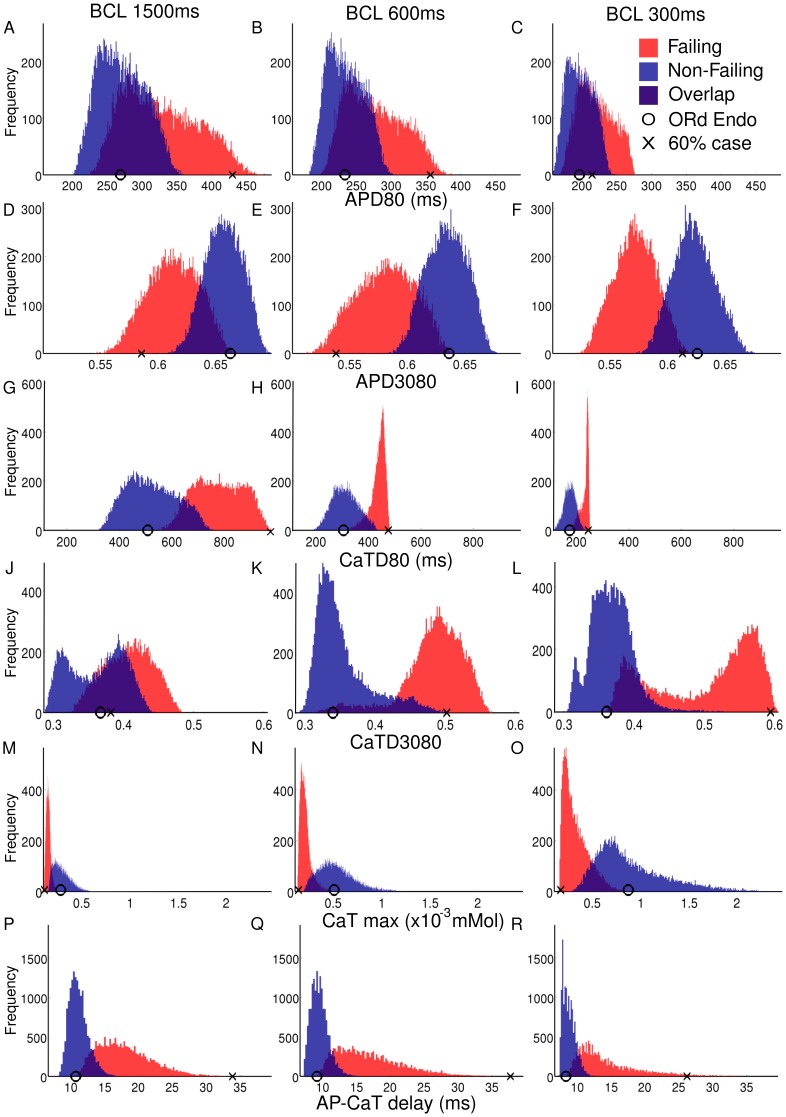
Histograms showing biomarker values obtained for the non-failing (blue) and failing (red) cell model populations. Histograms shown are recorded at BCL = 1500 ms, 600 ms and 300 ms (A–C: APD80, D–F: APD3080, G–I: CaTD80, J–L: CaTD3080, M–O: CaTmax and P-R: AP-CaT delay. In each panel, the circle indicates biomarker values obtained with the standard ORd model whereas the cross indicates the value obtained with the ORd model including 60% downregulation of G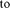, G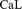, J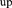 and G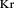 and 60% upregulation of G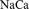.

Fig. 2 shows sample AP and Ca transients from the non-failing and failing populations at BCLs of 1500 ms and 300 ms. Histograms based on these traces, shown in Fig. 3, illustrate the variability encountered across populations for each biomarker. Simulation results show significant differences in biomarker values between the failing and non-failing populations at all BCLs, but an overlap between the two populations also exists. The circle in each panel indicates the biomarker value obtained using the standard ORd model. The cross in each panel indicates the biomarker value obtained by including specific values of 60% downregulation of G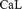, G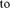, G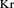 and J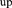 and 60% upregulation of G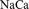 as indicated by the mean change in mRNA expression in Ambrosi *et al* [3] (therefore without considering electrophysological variability and uncertainty in ionic currents and electrophysiological measurements). As expected, biomarker values in the non-failing population are centered around results marked by the circle. In contrast, the cross is often located at the higher end of biomarker values for APD80, CaTD80, CaTD3080 and DAPCaT and at the lower end for APD3080, which could lead to an overestimation of remodeling-related changes. Results corresponding to each biomarker are discussed in detail below, and compared to previously published experimental studies, summarized in Table 2. A summary of the experimental data may be found in Tables S1–S7 in Supplement S1.

**Table 2:** Experimental data summary.

Biomarker	Increase in HF	Decrease in HF	No change in HF	Simulation 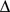 median (1500 ms BCL)
AP duration	[28], [29], [33], [27], [4](a), [6](a)		[5]	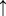 19.7%
AP triangulation	[28](b), [30](b)			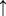 6.6%
CaT duration	[34], [28], [32], [33]		[5]	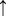 51.7%
CaT triangulation		[5]		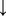 11.6%
CaT max		[28], [32], [33]	[31]	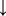 44.4%
AP-CaT delay	[34], [5]			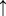 59.6%

a) [4] and [6] report a transmural flattening of APD in heart failure caused by prolongation of AP at the epicardium, but not in the midmyocardium or endocardium.

b) Not computed, see figures in those papers.

#### AP duration

As shown in Fig. 3 A–C, APD80 values in the non-failing population range between 201 and 362 ms for BCL = 1500 ms and between 158 and 248 ms for BCL = 300 ms. Introducing mRNA expression changes associated with heart failure results in prolonged APD80 values in the failing population with a 19.6% median increase at all BCLs. APD80 values in the failing population range between 223 ms and 486 ms for BCL = 1500 ms and between 167 ms and 277 ms for BCL = 300 ms. An overlap in APD80 values between the non-failing and failing populations exists for all BCLs.

As shown in Table S2 in Supplement S1, experimental recordings obtained in failing and non-failing cardiac preparations show a wide range of APD80 values, possibly explained by inter-subject and intra-subject variability as well as differences in experimental conditions and preparations between studies. Overall, experimentally-reported APD values are longer in failing hearts than in non-failing hearts, as our simulation results show in Fig. 3 A–C. In particular, our results for APD80 are comparable to those of [26], [27]. Both populations have significantly smaller absolute values than those published by Bueckelmann *et al* [28], [29], however these values are significantly longer than those reported in the other experimental studies we reviewed. Optical mapping measurements in the left-ventricular transmural wedge report AP prolongation at the epicardium and shortening at the endocardium in failing versus non-failing hearts, indicating potential location specific remodeling effects caused by heart failure [5].

#### AP triangulation

As shown in Fig. 3 D–F, AP triangulation increases (i.e. APD3080 decreases) by a median value of between 7 and 10% at all BCLs in the failing population as compared to the non-failing population, due to a decrease in APD30 relative to APD80. The range in APD3080 values observed in the failing population is larger than that observed in the non-failing population. AP triangulation was not generally quantified in the reviewed literature. However, visual inspection of APs given in experimental studies [28], [30] does reveal a marked increase in AP triangulation in failing myocytes, in agreement with our simulation results.

#### Calcium transient duration

CaTD80 is increased at all BCLs compared with the non-failing population (Fig. 3 G–I). At 1500 ms cycle length, the median value of CaTD80 in the failing population is 777 ms, a 51.7% increase in the median value recorded for the non-failing population of 512 ms. The percentage increase in the median value of the failing population decreases with cycle length to a 36% increase compared to the non-failing median value of 176 ms at a 300 ms cycle length. In agreement with our findings, prolongation of CaT was consistently reported in the reviewed literature, as summarized in Table 2. Numerical values from experimental studies are shown in full in Table S3 in Supplement S1.

#### Calcium transient triangulation

As shown in Fig. 3 J–L, CaTD3080 values tend to increase in the failing population relative to the non-failing population, corresponding to a decrease in the CaT triangulation. This is in agreement with the only experimental study to explicitly report CaT triangulation values by [5] (see Table S6 in Supplement S1).

Our simulations show that CaTD3080 values display a complex behaviour that is dependent upon the BCL in both the non-failing and failing populations. At 1500 ms BCL, CaTD3080 values for the non-failing population display a bimodality in their distribution corresponding to two subpopulations of cells, the larger one close to the value shown by the original ORd model, and the other one with markedly reduced CaTD3080 values. The latter disappears with decreasing BCLs below 1000 ms in the non-failing population. In the failing population however, the bimodality appears for BCLs 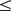 600 ms, with one sub-population appearing close to the original ORd model value and one significantly greater, corresponding to significantly decreased triangulation. At a BCL of 300 ms, the subpopulation with lower CaTD3080 values is a larger proportion of the failing population.

#### Maximum cytosolic calcium concentration

Fig. 3 M–O show that at all BCLs, both magnitude and range of CaTmax values decrease in the failing population as compared to the non-failing population. As BCL decreases, the range in values increases in each population. At 1500 ms the non-failing population has a median value of 2.82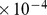 mM, increasing to 9.0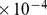 mM at 300 ms cycle length. The failing population has a median value of 1.58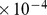 mM at 1500 ms, increasing to 2.96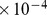 mM at 300 ms cycle length. In both populations, the dispersion increases as BCL decreases. The experimental values for maximum cytosolic calcium vary widely in the literature, but a reduction in failing hearts is reported consistently in line with our simulations (see Table S5 in Supplement S1). One potential disagreement with the experimental data is that Table S5 in Supplement S1suggests that systolic Ca may decrease with BCL contradicting what we see in our simulations. As this trend for decreasing systolic Ca with decreasing BCL spans multiple papers [31]–[33] there is a strong possibility that differences in preparation of myocytes and recording technique are responsible for this apparent effect.

#### AP-calcium transient delay

As illustrated in Fig. 3 P–Q, at all BCLs, DAPCaT values tend to be larger and more variable in the failing population than in the non-failing population, in agreement with the only experimental study to measure the AP-CaT delay [5]. Increased rise time to peak, as observed in Gwathmey *et al* [34] and Lou *et al* [5] is also consistent with slowed rise of the CaT, as shown in our simulations (Table S7 in Supplement S1).

At 1500 ms the median value for DAPCaT in the failing population is 17.4 ms, as opposed to 10.9 ms in the non-failing population. At a cycle length of 300 ms, the median value for the failing population is 13.1 ms and the median value for the non-failing population is 8.6 ms.

### Correlation between Biomarkers

**Figure 4:**
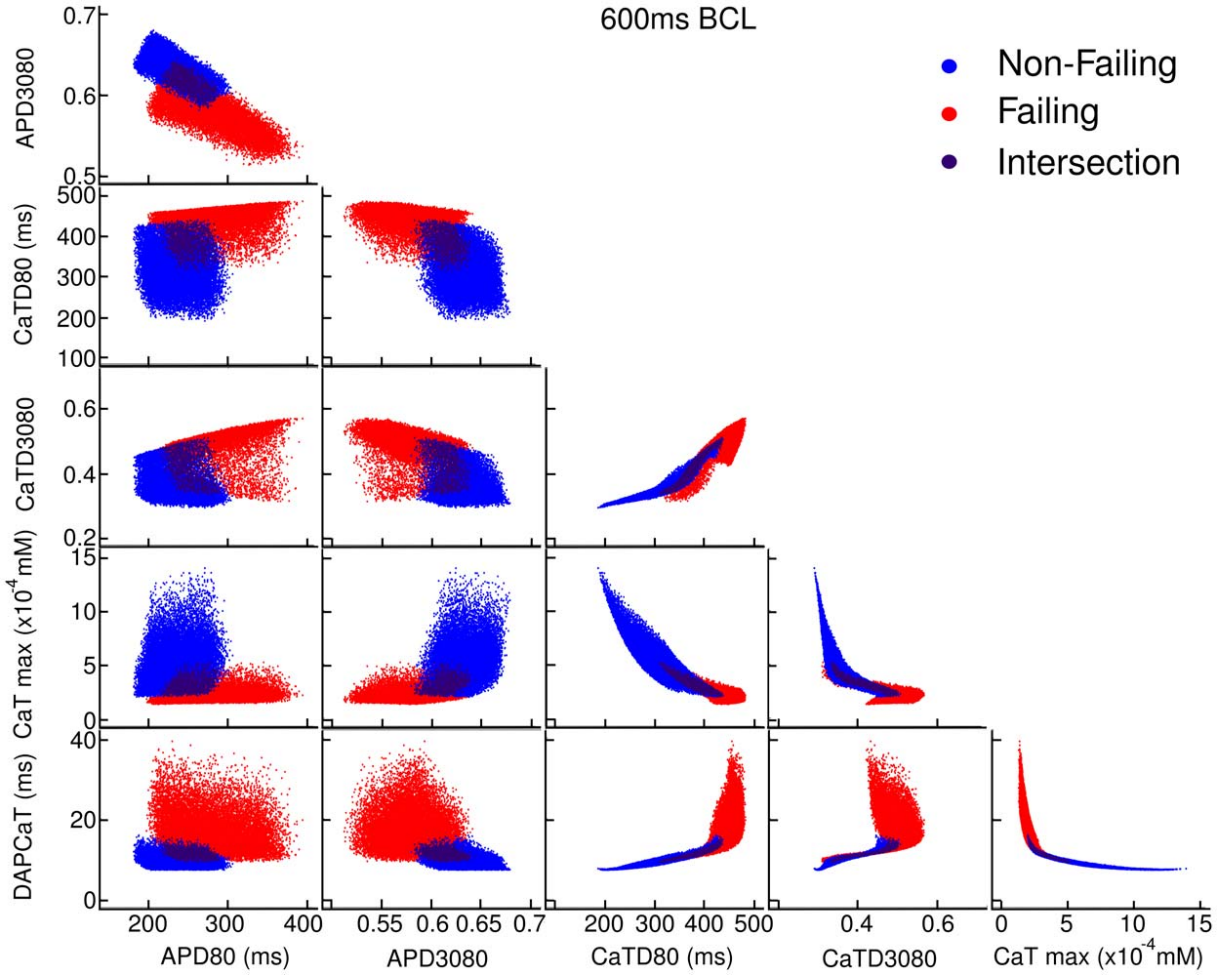
Correlation plots of one biomarker against another at 600 ms BCL. Red denotes the failing population, blue denotes the non-failing population, and the darker shade represents cells lying in the intersection between the two populations. Points which develop alternans are removed from these plots. Each biomarker is plotted against each other.

In order to determine the potential correlation between the different biomarkers values shown above, Fig. 4 plots values for each biomarker against each of the other biomarker values for each model in the non-failing and failing populations. As shown in Fig. 4 (first column, first row), APD80 and APD3080 are correlated as expected from the formulation of APD3080 and increasing APD80 is associated with increased triangulation (decreased APD3080) in both populations. The correlation is found for all BCL, except for BCL = 300 ms, where the failing population shows no such correlation (not shown). In general, AP biomarkers are only weakly correlated with CaT biomarkers suggesting the underlying ionic mechanisms driving changes in these biomarkers are different. The exception is APD80 at 1500 ms BCL in the failing population, which shows a correlation with CaTD3080.

Fig. 4 indicates that calcium biomarkers (CaTD80, CaTD3080, CaTMax and DAPCaT) show strong correlation with one another. In general, increased CaTD80 is correlated with decreased CaT triangulation (increased CaTD3080), decreased CaTmax and increased DAPCaT. Increased triangulation (decreased CaTD3080) is also correlated with decreased CaTmax, and increased DAPCaT in the non-failing population only. Finally increased CaTmax is correlated with decreased DAPCaT. The correlation shown between certain biomarkers may be explained by common ionic mechanisms, as explored below using regression analysis.

### Regression Analysis

**Figure 5:**
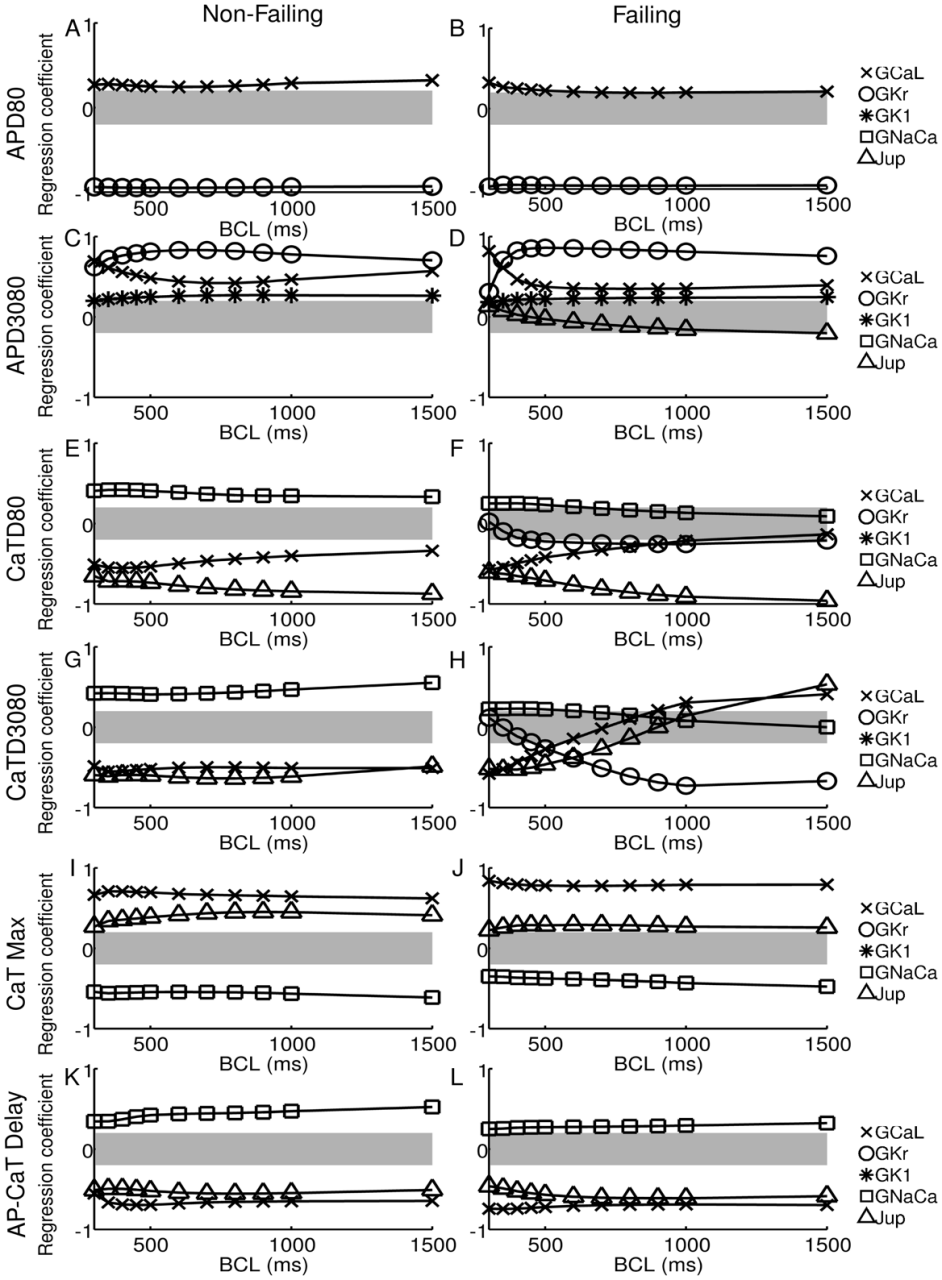
Regression coefficients obtained in the non-failing and failing populations. Coefficients are plotted at all cycle lengths for A,B) APD80, C,D) APD3080, E,F) CaTD80, G,H) CaTD3080, I,J) CaT max and K,L) AP-CaT delay. For clarity, only regression coefficients with an absolute value greater than 0.2 are plotted, denoted by the grey band.

We further exploit the potential of a population-based computational approach to investigate the relative importance of ionic properties in determining changes in each biomarker in failing versus non-failing human cardiomyoctes. The correlation found in the previous section with respect to AP and Ca biomarkers suggests that different ionic properties determine AP and Ca biomarkers values, respectively, within each population. We use regression analysis to establish which ionic currents determine biomarker values in the failing versus non-failing population. Fig. 5 shows regression coefficients for each of the biomarkers under investigation at all BCLs in the non-failing (left) and failing (right) populations. For clarity, only coefficients reaching an absolute value greater than 0.2 are given (those that come outside of the grey band in the plots). Regression coefficients are shown for both failing and non-failing populations. Examples of regression fits are shown in Fig. S1 A-D in Supplement S1. Regression coefficients for all biomarkers at all BCLs are shown in Tables S8–S19 in Supplement S1. The regression method produces good fits as measured by the 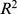 values which lay in a range between 0.75 and 0.98, with the exception of CaTD80 in the failing case. 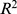 values for all regression fits are shown in Tables S20 and S21 in Supplement S1.

In both the failing and non-failing populations, long APD80 values are correlated with low G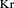, as shown by the large negative correlation coefficient of around −0.9 (Fig. 5A,B). Regression analysis also gives a small positive correlation of APD80 with G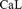 in the failing population that increases as BCL is decreased. In the non-failing population the effect of G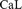 shows no significant change with BCL.

As expected from the strong correlation between APD80 and AP triangulation shown in Fig. 4, large AP triangulation (i.e. small APD3080 value) is correlated with low G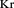, but unlike APD80 also with lowered G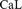, represented by the positive correlation coefficients shown in Fig. 5C,D. Lowered G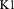 also makes a small contribution to the increase in triangulation. In both populations, the ionic mechanisms determining APD3080 are heavily rate dependent, with G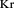 being the most important factor at long BCLs. At short BCLs of 350 ms and 300 ms AP morphology is driven primarily by G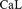, especially in the failing population.

As illustrated in Fig. 5 E–L, CaTD80, CaTD3080, CaTmax and DAPCaT are determined by J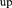, G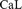 and G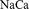 in both populations. This is consistent with their sharing the same underlying mechanisms determining these properties, as suggested by the strong correlations shown in Fig. 4. G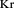 also affected CaTD3080 at longer cycle lengths in the failing population. Negative correlation coefficients of J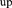 and G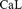 for CaTD80, CaTD3080 and DAPCaT indicate that low J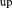 and G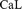 result in prolonged and less triangular CaTs with increased delay between the upstrokes of voltage and calcium. Positive correlation coefficients of J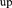 and G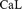 for CaTmax suggest that low Ca systolic levels in the failing population are determined by small J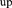 and G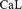. For each of the calcium transient biomarkers, the action of J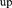 and G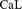 was opposed by that of G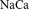. Despite being upregulated in heart failure, G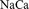 had a relatively smaller importance on biomarker values in the failing population than in the non-failing population. The relative importance of J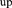 and G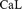 is rate dependent, with G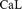 becoming increasingly important at shorter BCLs, as for AP triangulation. Note that the failing population shows a very different cause for low triangulation at long BCLs, with a negative correlation coefficient suggesting that decreased G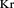 increases CaTD3080 and hence decreases triangulation, the opposite result to that for APD3080.

Even when 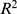 coefficents are low, regression analysis can be useful for identifying markedly non-linear responses in regions of parameter space. The concomitant effects of the parameters J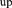 and G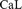 explain both the bimodality shown in Fig. 3 J–L and the low 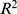 values for CaTD3080 regression fits. The non-linear relationship between J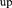 and G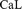 (as shown in Fig. S2 and discussed in Supplement S1) results in the majority of the parameter space giving either a high or low value for this biomarker. A relatively small region of parameter space separates the two regions, indicating that a large change in either of these parameters individually could produce similar effects in morphology to a smaller change in both parameters simultaneously.

### Alternans

**Figure 6:**
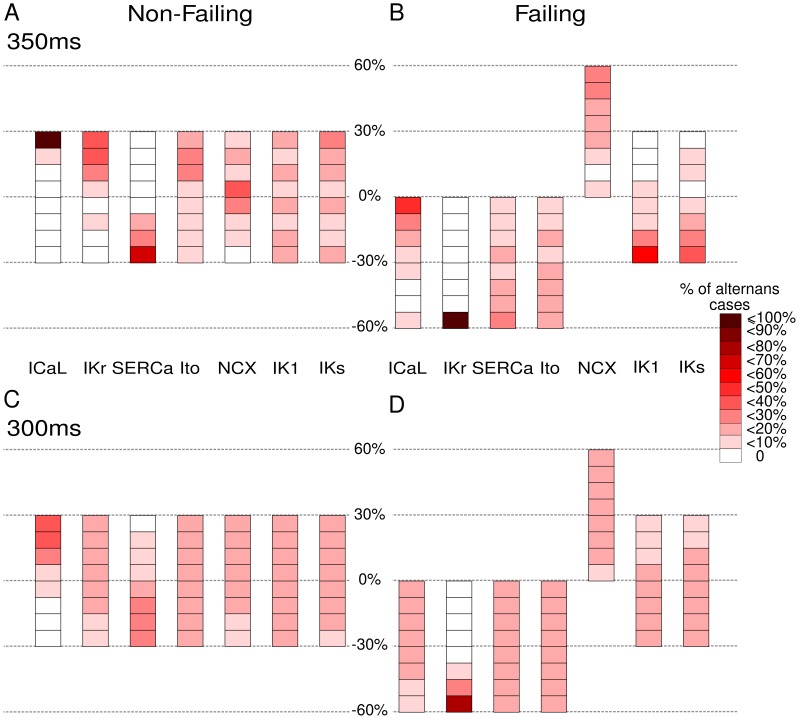
Occurrence of voltage and calcium alternans in the non-failing and failing populations. Colour scale indicates the proportion of models displaying alternans in APD80 and/or CaTD80 for each value of each ionic conductance considered. A) There are 36 non-failing alternans cases at 350 ms BCL. B) There are 64 failing alternans cases at 350 ms BCL. C) There are 2241 non-failing cells in alternans at 300 ms BCL. D) There are 2235 failing cell models in alternans at BCL 300 ms.

Fig. 6 depicts the number of models displaying alternans in both populations together with a heat map of the parameters used to generate them. In the non-failing population beat-to-beat alternans are observed in the amplitude of the calcium transient and in the APD as BCL is decreased. Alternans onset begins in 36 non-failing cells at BCL 350 ms and is apparent in 2241 of the non-failing cells by BCL 300 ms. In the standard ORd model, alternans are not observed until BCL 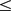 300 ms. 2∶1 block is reported at BCL 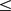 250 ms for the standard ORd model by [15]. As can be seen in Fig. 6 A,C alternans occur in non-failing cell models with a combination of low J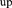 and high G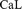.

In the failing population alternans occur in 64 cells at 350 ms and in 2235 cells at a BCL of 300 ms, due to prolonged APD associated with low G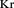 (Fig. 6 B,D), as demonstrated by the regression analysis. Representative voltage and calcium traces for both populations are shown in Fig. S3 in Supplement S1.

## Discussion

In this study we have developed a population-based approach to investigate the potential of mRNA expression levels measured in failing human hearts to predict electrophysiological remodeling in AP and CaT, as measured experimentally. Our population-based approach takes into consideration natural variability that may arise from a variety of sources, as well as uncertainty associated with the extent to which mRNA expression affects functional channel conductance at the cell membrane, and uncertainty in electrophysiological measurements. This is in contrast to the traditional approach of considering unique values of ionic conductances in cardiac models of non-diseased and failing human cardiomyocytes [15], [30], [35]. Our main findings are:

1. mRNA data associated with G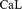, G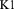, G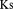, G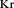, SERCA, G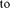 and G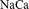 (as reported in [3]) predict prolongation and increased triangulation of AP, prolongation and decreased triangulation of Ca transients, decreased maximum systolic Ca, and increased delay between Ca and Vm upstroke in failing versus non-failing human cardiomyocytes, as reported in experimental studies (Tables 2 and S1–S7 in Supplement S1);
2. AP biomarkers (namely APD80 and APD3080) are correlated in both non-failing and failing cardiomyocytes, and are primarily determined by G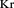, and secondarily by G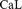 and G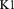;
3. Ca biomarkers (including CaTD80, CaTD3080 and DAPCaT) are correlated (but only weakly correlated with AP biomarkers) and are primarily determined by J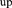, G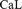 and G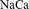;
4. ionic mechanisms underlying the occurrence of alternans are different in the failing population versus the non-failing population: alternans are primarily favoured by low G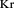 values in failing human cardiomyocytes and by low SERCA (J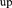) and high G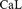 in non-failing human cardiomyocytes.

The non-failing and failing populations of human ventricular cell models are constructed based on the latest human ventricular AP model [15], which was developed using a consistent and extensive dataset of electrophysiological measurements obtained from the left ventricular endocardium of non-diseased human hearts. The non-failing cell model population includes human ventricular cell models sharing the same equations but with ionic conductances varying in 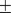
30% range with respect to control. The overlap in the parameter ranges between the two populations explains some (but not all) of the overlap in the histograms in Fig. 3. Variability in ionic conductances lead to variability in biomarker values, with each distribution centered around the original ORd model value. Specifically, APD80 ranges from 201 ms and 362 ms for BCL = 1500 ms and from 158 ms and 248 ms for BCL = 300 ms, which is in range with the level of variability reported in experimental measurements for non-diseased human cardiomyocytes [15], [16]. As shown in Table S2 in Supplement S1, APD values are very different from one study to another, indicating important sources of variability related to preparations and techniques specific to each study. This may represent an important limitation for the construction of cardiac models built using a variety of data from different laboratories [36].

The failing cell model population is built by including 0–60% downregulation of G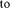, G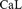, G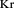 and SERCA and 0–60% upregulation of I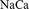, based on the data by [3]. Our simulations predict changes in AP and Ca transients, which are in qualitative agreement with experimental recordings summarized in Tables S2–S7 in Supplement S1. Significant quantitative differences exist between experimental studies and also with our simulation results, which might be due to a range of causes including the type of heart failure (non-ischæmic versus ischæmic), inter-subject variability, preparation (isolated cells or tissue), recording technique, etc [37].

Changes in AP biomarkers are not strongly correlated with changes in CaT biomarkers, suggesting that different ionic mechanisms cause these biomarker changes. This is explicitly demonstrated through linear regression. In particular different causes determine the duration of AP and CaT in failing cardiac myocytes, namely G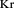 for the AP, and SERCA and G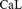 for the Ca transient. In the reviewed comparative experimental studies, AP and CaT prolongation occurred concurrently in heart failure. Thus, our findings lead to the prediction that remodeling of both calcium and potassium currents currents may have occurred in these samples - remodeling of one component alone does not lead to the changes seen in these experiments. It is worth noting that [5] observed no significant prolongation of APD80 or CaTD80 at the endocardium in the failing population, but did observe other changes in CaT morphology. We suggest that correlation plots may provide a method to investigate causes of biomarker changes by plotting against biomarkers whose underlying regulatory mechanisms are well known.

Our simulations also show significant rate-dependence in the ionic conductances underlying changes in AP triangulation, CaTD80, CaT triangulation and AP-CaT delay in failing and non-failing cardiomyocytes. In particular, the significance of I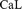 in affecting the biomarkers under investigation increases at shorter cycle lengths.

Our population based approach reveals conditions in which alternans may appear at longer BCLs than in the original ORd model. The form of alternans observed in the non-failing population cannot occur in the failing population, as the combination of parameters driving alternans in the non-failing population is outside the parameter range for the failing population. Similarly, the 2∶1 block observed in the failing population does not occur in the non-failing population as G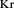 cannot attain sufficiently low values. Thus, the alternans effect is different in each population, arising from different ionic mechanisms. This suggests that alternans observed in failing and non-failing hearts may have different causes, and could require different interventions to correct.

In this study we have only examined one component of the mRNA expression dataset presented by Ambrosi *et al* [3], namely the changes that occur between non-failing and failing human left ventricle. There are several other avenues of investigation still open from this dataset, namely atrial differences and gender specific differences. Whilst Ambrosi *et al* [3] noted no significant difference between male and female left ventricles, other studies have done so [1], [14]. This novel population-based approach to modelling disease states is a first step towards clinically relevant modelling of disease states as the methodology is flexible enough to be applied to any dataset in order to utilise the information extracted from mRNA data.

### Limitations

The non-failing human hearts studied are not necessarily healthy, and so care must be taken in extrapolation from these results to healthy humans [5]. Similarly, the term ‘heart failure’ describes a range of pathologies as shown in Table S1 in Supplement S1, and so our results may not extend to all conditions coming under the description ‘heart failure’. For example, Ambrosi *et al* [3] reported a downregulation of NCX1 in non-ischæmic but not ischæmic cardiomyopathy as compared to non-failing hearts (see Table 1).

We do not include co-regulation of membrane ion channels in our study, as this information is not available from Ambrosi *et al* [3]. Co-regulation could have an effect upon the choice of distribution used for the parameters in future studies.

The presence of spatial heterogeneities in AP properties in the left ventricle is consistently reported in humans [4], [38]. We do not directly address these heterogeneities in our study, although our population approach does offer robustness against variability resulting from this. Furthermore, many experimental studies use isolated ventricular myocytes whose original location within the heart is unclear. The left ventricular wedge studies [4]–[6] go some way to addressing this issue, however intercellular coupling may also affect action potentials as compared to isolated cells.

Our study inherits many of the difficulties in extrapolating from mRNA expression data to functional membrane ion channels as discussed in the introduction and reviewed in [11]. Furthermore, we explicitly address only changes in conductance, and do not consider other potential consequences of heart failure, such as altered gating dynamics [39].

## Supporting Information

Supplement S1 **Supplementary figures and tables referred to in the text.**
(PDF)
