# Supplementary material for: mRNA Expression Levels in Failing Human Hearts Predict Cellular Electrophysiological Remodeling: A Population-Based Simulation Study
Source: PLoS One. 2013 Feb 20;8(2):e56359. doi: 10.1371/journal.pone.0056359 (PMC3577832; doi:10.1371/journal.pone.0056359)
Supplement: Supplement S1 — Supplementary figures and tables referred to in the text. (PDF) [file pone.0056359.s001.pdf]

mRNA expression levels in failing human hearts predict cellular electrophysiological remodelling: A population-based simulation study

Supplementary material

Linear regression and CaTD3080

It can be seen from Tables S20 and S21 that the linear regression fits obtained to data were in general of a high quality. The exception was the case of CaTD3080, where linear regression scores were low. The reasons for this can be seen in Fig. S1, which demonstrates the failure of the linear model in the case of this biomarker. The reason for this is that in the case of CaTD3080, Jup and GCaL have a nonlinear relationship which causes the linear model to fail as shown in Fig. S2. However, the regression coefficients do still correctly identify the parameters which cause the largest changes in this case, and so are still included in the analysis. Note that this need not always be the case. We do not claim in this case that the linear fit is accurate, but instead that the regression coefficient indicates which parameters demonstrate the largest cause of change in the population. This finding demonstrates that

1. Results of regression analysis must always be checked against actual parameter outputs;

2. Linear regression analysis will not be appropriate for parameterising models as proposed in [1, 2] in all cases.

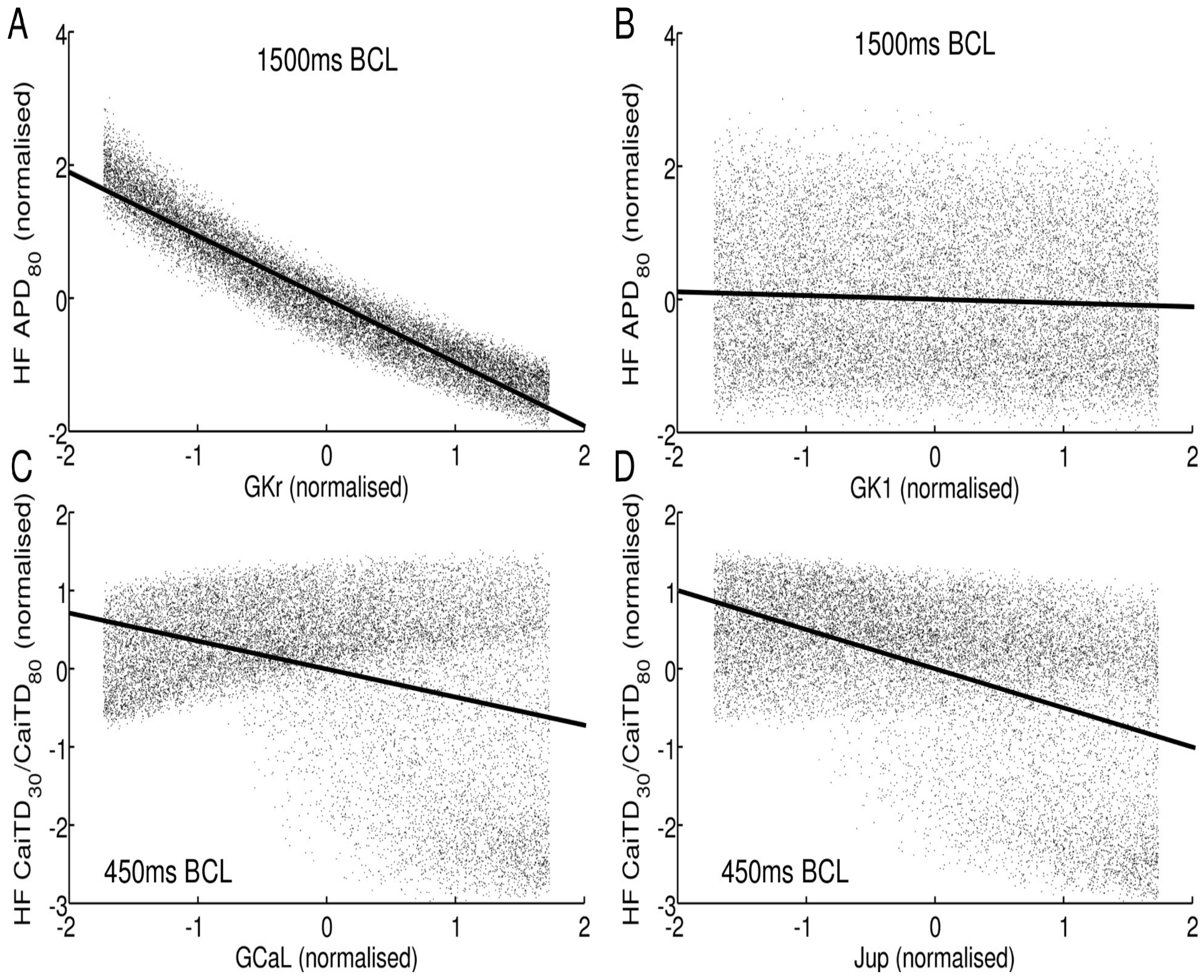

Figure S1. Linear regression lines for individual parameters. Regression lines plotted over the data set for A) GKr and B) GK1 in the HF population at BCL 1500ms, and for C) GCaL and D) Jup in the failing population at BCL 450ms to illustrate low R^2 in this case.

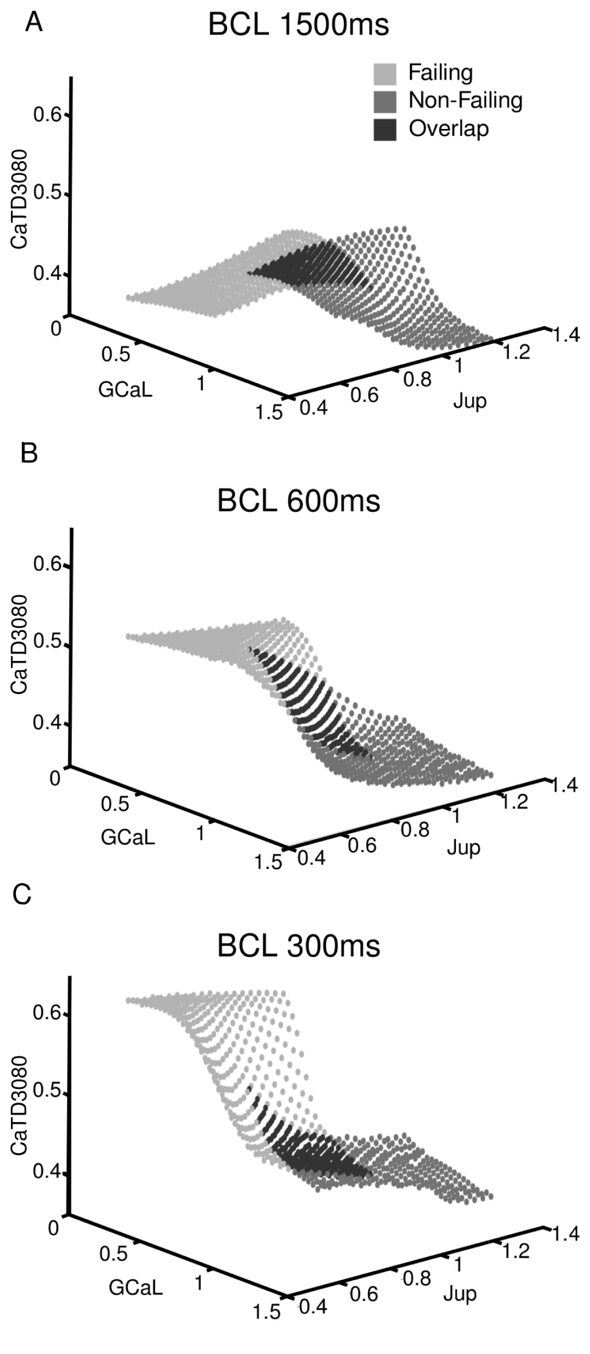

Figure S2. Parameter surfaces for CaT3080 as a function of GCaL and Jup. The parameter surfaces were generated by allowing only GCaL and Jup, then plotting both F and NF populations together. Shown are A) BCL 1500ms, B) BCL 600ms and C) BCL 300ms.

Representative traces in alternans cases

In Fig. S3 we show two sample voltage and calcium traces from both the failing and non-failing populations in order to illustrate the form of alternans observed. The cell model number is the number of the experiment within the failing or non-failing experimental designs as appropriate. These are available to download from `http://www.cs.ox.ac.uk/chaste` along with all code as mentioned in the main article.

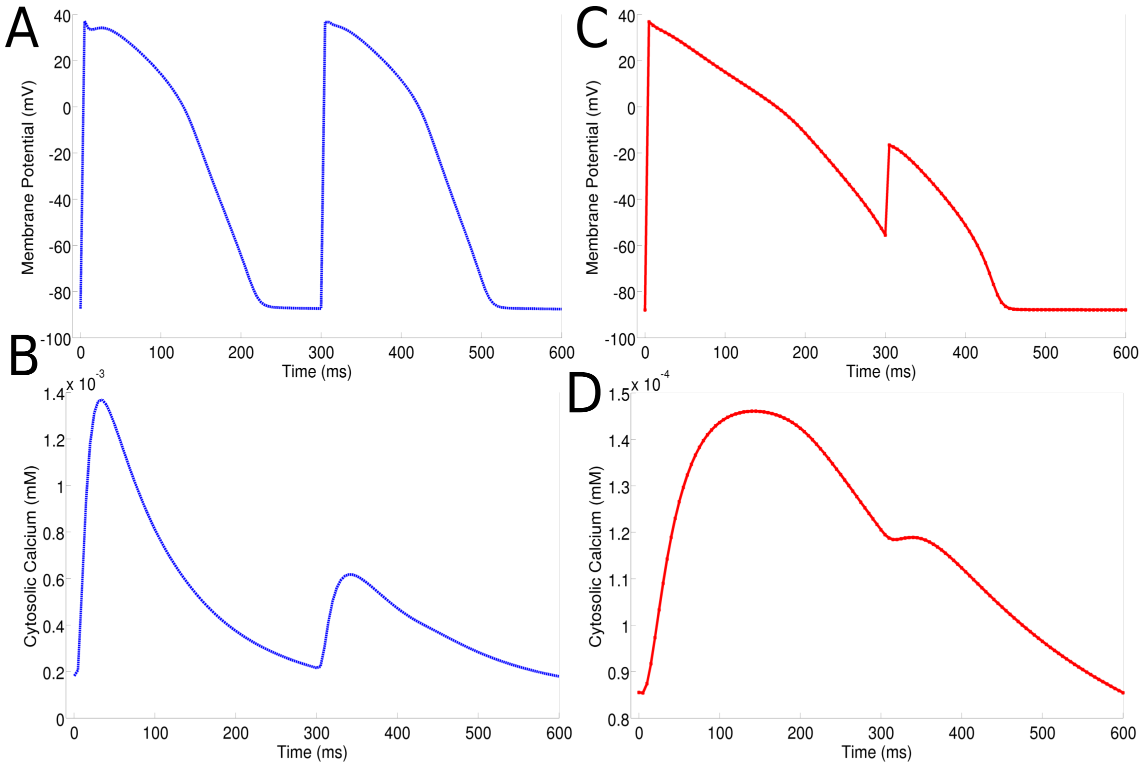

Figure S3. Alternans traces in the non-failing population (blue) and the failing population (red). Voltage A) and calcium B) traces for cell NF #6778 at BCL 300ms. Conductivity scales are: G_{CaL} 1.2554, G_{Kr} 1.1231, J_{up} 0.8646, G_{to} 1.0349, G_{NaCa} 1.1225, G_{K1} 0.9713, G_{Ks} 1.0232. Voltage C) and calcium D) traces for cell F #9441 at BCL 300ms. Conductivity scales are: G_{CaL} 0.4025, G_{Kr} 0.4280, J_{up} 0.9912, G_{to} 0.8149, G_{NaCa} 1.5197, G_{K1} 1.0540, G_{Ks} 0.8976.

Table S1. Experimental Data: Population Details

Publication	Preparation	Recording Method	Cohort	Gender	Age
Gwathmey *et al* 1987 [3]	RV muscle strips	Aequorin	8 NF	3 f, 5 m	7-56
			9 F (3 Id, 4 Is, 1 MD, 1 NDBVH)	2 f, 5 m	14-54
Beuckelmann *et al* 1992 [4]	isolated LV myocyte	Fura-2, patch clamp	3 NF	not given	not given
			14 F (9 D, 5 Is)	3 f, 11 m	51.8+/-11.6
Beuckelmann *et al* 1993 [5]	isolated LV myocyte	patch clamp	6 NF	not given	not given
			16 F (10 D, 6 Is)	2 f, 14 m	46.6+/-3.6
Vahl *et al* 1994 [6]	LV muscle strips	Fura-2	3 NF	not given	not given
			7 F (7 D)	not given	not given
Sipido *et al* 1998 [7]	isolated LV myocyte	Fluo 3 (+Fura red),	5 F (Is)	5 male	48-67
		patch clamp	6 F (D)	3 f, 3 m	21-60
Kubo *et al* 2001 [8]	isolated LV myocyte	Fluo 3+voltage clamp	10 NF	not given	56.3+/-4.4
			20 F (10 IsD, 10 NIsD)	not given	IsD 56.4+/-3.7, NIsD 48.6+/-4.4
Piacento *et al* 2003 [9]	isolated LV myocyte	Fluo 3+ AP clamp,	7 NF	2 f, 5 m	66+/-3
		patch clamp	11 F (5 Is, 6 IdD/NIsD)	2 f, 9 m	49+/-4
Li *et al* 2004 [10]	isolated RV myocyte	patch clamp	5 NF	not given	30-65
			6 F	not given	34-58
Glukhov *et al* 2010 [11]	transmural LV wedge	di-4-ANEPPS	5 NF	2 f, 3 m	19-68
			5 F (1 Id, 1 Is, 1 R, 1 NIs, 1 D)	1 f, 4 m	28-64
Lou *et al* 2011 [12]	transmural LV wedge	RH237. Rhod-2 AM	6 NF	2 f, 3 m	47-59
			5 F (2 Is, 3 Id)	3 f, 2 m	49-65
Glukhov *et al* 2012 [13]	transmural LV wedge	di-4-ANEPPS	10 NF	5 f, 5 m	19-76
			10 F (4 Id, 3 D, 2 NIs, 1 H)	6 f, 4 m	28-66

Abreviations used: F = failing, NF = non-failing, D = dilated cardiomyopathy, Is = ischaemic cardiomyopathy, MD = myocardial deterioration post-surgery, NDBVH = non-dilated biventricular hypertrophy, IsD = Ischaemic dilated cardiomyopathy, NIsD = non-ischaemic dilated cardiomyopathy, IdD = idiopathic dilated cardiomyopathy, NIs = non-ischaemic cardiomyopathy, R = restrictive cardiomyopathy, H = hypertrophic cardiomyopathy, f = female, m = male. Ages are displayed as either mean +/- standard deviation or min-max.

Table S2. Experimental Data: Action potential biomarkers

Publication	BCL	Cohort	APD90(ms)	APD80(ms)				APD50 (ms)
				endo	mid	epi	max	
Beuckelmann *et al* 1992 [4]	2000ms	NF	649+/-101					
		F	1038+/223					
Beuckelmann *et al* 1993 [5]	2000ms	NF	650+/-100					
		F	1090+/-90					
Sipido *et al* 1998 [7]	4000ms	F (Is)	537+/133					433+/-31
		F (D)	516+/-49					408+/-45
	2000ms	F (Is)	496+/-27					389+/-29
		F (D)	474+/-41					369+/-40
	1000ms	F (Is)	451+/-19					343+/-7
		F (D)	417+/-31					311+/-29
	500ms	F (Is)	352+/-19					255+/-17
		F (D)	341+/-20					250+/-23
Piacentino *et al* 2003 [9]	2000ms	NF						511+/-49
		F						663+/-29
Li *et al* 2004 [10]	2000ms	NF	364+/-23					241+/-22
		F	545+/-51					397+/-42
	1000ms	NF	305+/-17					234+/-16
		F	438+/-38					305+/-28
	500	NF	281+/-16					213+/-12
		F	324+/-19					234+/-19
Glukhov *et al* 2010 [11]	4000ms	NF		510+/-22	471+/-21	408+/-21	615+/-45	
		F		516+/-39	507+/-32	482+/-25	521+/-30	
	2000ms	NF		494+/-22	455+/-20	383+/-21	537+/-40	
		F		506+/-35	495+/-25	477+/-22	495+/-30	
	1000ms	NF		437+/-18	405+/-16	350+/-16	466+/-25	
		F		450+/-35	437+/-31	415+/-18	455+/-34	
	750ms	NF		383+/-21	362+/-20	316+/-16	410+/-16	
		F		373+/-21	367+/-19	357+/-13	379+/-20	
	500ms	NF		316+/-15	297+/-12	263+/-12	330+/-12	
		F		305+/-17	299+/-13	292+/-9	313+/-11	
	300ms	NF		226+/-9	216+/-8	196+/-8	228+/-3	
		F		207+/-1	206+/-6	196+/-5	215+/-8	
Lou *et al* 2011 [12]	1500ms	NF		451+/-85	420+/-81	394+/-81		
		F		426+/-70	408+/-64	400+/-66		
	1000ms	NF		398+/-62	380+/-61	361+/-47		
		F		394+/-65	375+/-50	364+/-50		
	800ms	NF		359+/-60	348+/-53	330+/-56		
		F		353+/-43	338+/-44	329+/-46		
	600ms	NF		315+/-43	303+/-37	294+/-44		
		F		308+/-43	291+/-38	295+/-43		
Glukhov *et al* 2012 [13]	4000ms	NF		492+/-18	453+/-17	387+/-19		
		F		561+/-35	538+/-26	512+/-29		
	2000ms	NF		481+/-17	441+/-16	371+/-16		
		F		525+/-35	481+/-31	468+/-27		
	1000ms	NF		429+/-14	398+/-13	342+/-13		
		F		439+/-15	413+/-13	386+/-15		
	750ms	NF		374+/-16	351+/-16	305+/-13		
		F		372+/-14	352+/-13	328+/-16		
	500ms	NF		311+/-11	292+/-9	256+/-9		
		F		319+/-18	304+/-10	280+/-10		
	300ms	NF		226+/-6	212+/-5	192+/-6		
		F		21+/-9	202+/-7	194+/-11		

Abreviations used: F = failing, NF = non-failing, Is = ischaemic cardiomyopathy, D = dilated cardiomyopathy. Max denotes the maximum APD value in the tissue. All values given as mean +/- standard deviation.

Table S3. Experimental Data: Calcium transient durations

Publication	BCL	Cohort	CaTD80 (ms)				CaTD50 (ms)
			-	endo	mid	epi	
Gwathmey *et al* 1987 [3]	3000ms	NF	246+/-37				
		F	546+/-48				
Beuckelmann *et al* 1992 [4]	2000ms	NF					320+/-68
		F					692+/-166
Kubo *et al* 2001 [8]	500ms	NF					555+/-20
		F					664+/-42
Lou *et al* 2011 [12]	1500ms	NF		530+/-80	486+/-69	446+/-73	
		F		538+/-88	493+/-71	465+/-75	
	1000ms	NF		475+/-58	441+/-56	414+/-60	
		F		489+/-81	449+/-64	436+/-64	
	800ms	NF		441+/-43	410+/-46	376+/-49	
		F		435+/-59	412+/-48	395+/-45	
	600ms	NF		393+/-21	366+/-26	333+/-30	
		F		384+/-48	364+/-31	358+/-46	

Abreviations used: F = failing, NF = non-failing, - denotes no transmural spatial location given. All values given as mean +/- standard deviation.

Table S4. Experimental Data: Calcium transient decay time constants

Publication	BCL	Cohort	CaT τ (ms)			
			-	endo	mid	epi
Kubo *et al* 2001 [8]	500ms	NF	269+/-75			
		F	372+/-71			
Piacentino *et al* 2003 [9]	1000ms	NF	209+/-31			
		F	306+/-27			
Lou *et al* 2011 [12]	1500ms	NF		152+/-12	137+/-11	128+/-7
		F		168+/-22	154+/-21	142+/-13

Abreviations used: F = failing, NF = non-failing, τ = time constant of decay for the calcium transient, - denotes no transmural spatial location given.All values given as mean +/- standard deviation.

Table S5. Experimental Data: Calcium transient magnitude

Publication	BCL	Cohort	Diastolic Cai (nM)	Systolic Cai (nM)	Δ Cai (nM)
Beuckelmann *et al* 1992 [4]	2000ms	NF	96+/-47	746+/-249	
		F	165+/-61	367+/-109	
Vahl *et al* 1994 [6]	-	NF	195+/-80	1435+/-230	
		F	234+/-75	1562+/-172	
Kubo *et al* 2001 [8]	500ms	NF	125+/12	506+/-25	
		F	150+/-16	407+/-16	
Piacentino *et al* 2003 [9]	1000ms	NF	153+/-20		804+/-197
		F	147+/-14		398+/-58

Abreviations used: F = failing, NF = non-failing, Δ denotes change throughout CaT. All values given as mean +/- standard deviation.

Table S6. Experimental Data: Calcium transient triangulation

Publication	BCL	Cohort	CaTD30/CaTD80		
			endo	mid	epi
Lou *et al* 2011 [12]	1500ms	NF	0.53+/-0.03	0.50+/-0.03	0.45+/-0.05
		F	0.58+/-0.04	0.57+/-0.02	0.52+/-0.05

Abreviations used: F = failing, NF = non-failing. All values given as mean +/- standard deviation.

Table S7. Experimental Data: Calcium transient upstroke

Publication	BCL	Cohort	Time to peak(ms)*				AP-CaT delay (ms)		
			-	endo	mid	epi	endo	mid	epi
Gwathmey *et al* 1987 [3]	3000ms	NF	33+/-6						
		F	53+/-5						
Lou *et al* 2011 [12]	1500ms	NF		30+/-3	25+/-3	35+/-3	8.8+/-0.5	8.3+/-0.5	8.1+/-0.7
		F		38+/-16	27+/-4	27+/-6	9.7+/-0.5	9.0+/-0.2	8.0+/-0.4

Abreviations used: F = failing, NF = non-failing, - denotes no transmural spatial location given. All values given as mean +/- standard deviation. *Time to peak is given as the time from 10% to 90% rise time for the CaT in [12].

Regression coefficients

Table S8. Regression coefficients: nonfailing, APD80

Parameter	BCL (ms)									
	1500	1000	900	800	700	600	500	450	400	350	300
GCaL	0.322	0.289	0.269	0.256	0.247	0.244	0.250	0.256	0.267	0.278	0.270
GKr	-0.932	-0.937	-0.942	-0.945	-0.947	-0.948	-0.948	-0.948	-0.946	-0.943	-0.938
GKs	-0.078	-0.092	-0.093	-0.096	-0.099	-0.102	-0.106	-0.107	-0.108	-0.108	-0.106
GK1	-0.051	-0.054	-0.056	-0.058	-0.060	-0.062	-0.064	-0.065	-0.067	-0.071	-0.078
GNaCa	0.088	0.112	0.120	0.125	0.127	0.124	0.114	0.107	0.098	0.087	0.073
Gto	0.015	0.009	0.007	0.005	0.004	0.003	0.004	0.004	0.005	0.006	0.008
Jup	0.042	0.044	0.038	0.033	0.027	0.022	0.016	0.012	0.007	0.003	-0.003

Regression coefficients for the fit to APD80 in the non-failing population at each BCL.

Table S9. Regression coefficients: nonfailing, APD3080

Parameter	BCL (ms)										
	1500	1000	900	800	700	600	500	450	400	350	300
GCaL	0.570	0.465	0.437	0.422	0.422	0.441	0.483	0.519	0.558	0.610	0.692
GKr	0.705	0.778	0.801	0.820	0.832	0.832	0.814	0.792	0.764	0.724	0.618
GKs	-0.049	-0.057	-0.055	-0.052	-0.049	-0.045	-0.042	-0.039	-0.038	-0.042	-0.060
GK1	0.265	0.269	0.271	0.270	0.267	0.260	0.249	0.241	0.232	0.220	0.201
GNaCa	0.098	0.125	0.116	0.093	0.056	0.006	-0.053	-0.087	-0.115	-0.138	-0.152
Gto	0.176	0.158	0.150	0.142	0.132	0.121	0.111	0.104	0.099	0.098	0.102
Jup	0.063	0.024	0.009	0.007	0.017	0.041	0.076	0.096	0.115	0.132	0.140

Regression coefficients for the fit to APD3080 in the non-failing population at each BCL.

Table S10. Regression coefficients: nonfailing, CaTD80

Parameter	BCL (ms)										
	1500	1000	900	800	700	600	500	450	400	350	300
GCaL	-0.338	-0.403	-0.421	-0.440	-0.465	-0.497	-0.535	-0.547	-0.552	-0.548	-0.509
GKr	-0.029	0.002	0.017	0.031	0.045	0.056	0.060	0.058	0.048	0.032	0.030
GKs	-0.001	0.003	0.004	0.006	0.008	0.010	0.012	0.012	0.011	0.009	0.007
GK1	-0.003	-0.004	-0.004	-0.004	-0.004	-0.004	-0.004	-0.004	-0.004	-0.001	0.010
GNaCa	0.334	0.344	0.346	0.354	0.368	0.388	0.407	0.417	0.420	0.420	0.407
Gto	-0.036	-0.037	-0.036	-0.036	-0.036	-0.037	-0.038	-0.039	-0.039	-0.040	-0.038
Jup	-0.873	-0.843	-0.833	-0.820	-0.800	-0.771	-0.736	-0.721	-0.716	-0.718	-0.654

Regression coefficients for the fit to CaT80 in the non-failing population at each BCL.

Table S11. Regression coefficients: nonfailing, CaTD3080

Parameter	BCL (ms)									
	1500	1000	900	800	700	600	500	450	400	350	300
GCaL	-0.509	-0.508	-0.503	-0.498	-0.497	-0.504	-0.520	-0.532	-0.547	-0.553	-0.485
GKr	0.045	0.104	0.123	0.141	0.158	0.175	0.186	0.189	0.184	0.173	0.165
GKs	0.011	0.017	0.019	0.022	0.024	0.026	0.027	0.028	0.028	0.028	0.026
GK1	-0.005	-0.007	-0.006	-0.005	-0.004	-0.002	-0.001	-0.001	-0.002	-0.000	0.006
GNaCa	0.553	0.469	0.450	0.434	0.421	0.413	0.408	0.415	0.420	0.424	0.422
Gto	-0.051	-0.044	-0.040	-0.037	-0.035	-0.033	-0.033	-0.034	-0.035	-0.037	-0.036
Jup	-0.487	-0.615	-0.630	-0.637	-0.635	-0.622	-0.600	-0.593	-0.595	-0.610	-0.595

Regression coefficients for the fit to CaTD3080 in the non-failing population at each BCL.

Table S12. Regression coefficients: nonfailing, CaTmax

Parameter	BCL (ms)										
	1500	1000	900	800	700	600	500	450	400	350	300
GCaL	0.622	0.647	0.654	0.660	0.666	0.677	0.697	0.701	0.709	0.710	0.665
GKr	-0.117	-0.136	-0.146	-0.153	-0.155	-0.151	-0.135	-0.119	-0.095	-0.069	-0.051
GKs	-0.014	-0.019	-0.020	-0.022	-0.023	-0.024	-0.023	-0.021	-0.018	-0.015	-0.010
GK1	0.002	0.003	0.003	0.003	0.003	0.003	0.003	0.004	0.003	-0.000	-0.014
GNaCa	-0.612	-0.565	-0.554	-0.548	-0.545	-0.544	-0.544	-0.550	-0.554	-0.560	-0.542
Gto	0.056	0.053	0.052	0.051	0.050	0.050	0.050	0.050	0.051	0.053	0.050
Jup	0.412	0.450	0.453	0.449	0.437	0.416	0.386	0.372	0.356	0.341	0.271

Regression coefficients for the fit to CaTmax in the non-failing population at each BCL.

Table S13. Regression coefficients: nonfailing, AP-CaT delay

Parameter	BCL (ms)										
	1500	1000	900	800	700	600	500	450	400	350	300
GCaL	-0.645	-0.650	-0.653	-0.657	-0.664	-0.677	-0.695	-0.696	-0.689	-0.665	-0.553
GKr	0.110	0.114	0.119	0.123	0.125	0.122	0.107	0.091	0.069	0.062	0.090
GKs	0.012	0.015	0.016	0.016	0.018	0.018	0.018	0.016	0.015	0.013	0.019
GK1	-0.003	-0.003	-0.003	-0.003	-0.004	-0.004	-0.006	-0.009	-0.014	-0.005	0.008
GNaCa	0.524	0.472	0.458	0.449	0.443	0.437	0.423	0.403	0.371	0.346	0.344
Gto	-0.036	-0.036	-0.036	-0.036	-0.036	-0.036	-0.035	-0.034	-0.032	-0.029	-0.028
Jup	-0.508	-0.547	-0.553	-0.555	-0.552	-0.541	-0.521	-0.506	-0.494	-0.493	-0.510

Regression coefficients for the fit to AP-CaT delay in the non-failing population at each BCL.

Table S14. Regression coefficients: failing, APD80

Parameter	BCL (ms)									
	1500	1000	900	800	700	600	500	450	400	350	300
GCaL	0.212	0.201	0.197	0.197	0.201	0.210	0.225	0.238	0.252	0.267	0.324
GKr	-0.955	-0.957	-0.959	-0.959	-0.958	-0.957	-0.954	-0.952	-0.948	-0.947	-0.970
GKs	-0.086	-0.097	-0.098	-0.100	-0.101	-0.103	-0.103	-0.103	-0.102	-0.101	-0.102
GK1	-0.054	-0.057	-0.058	-0.060	-0.061	-0.062	-0.064	-0.066	-0.069	-0.075	-0.085
GNaCa	0.105	0.094	0.090	0.085	0.081	0.076	0.071	0.067	0.063	0.059	0.058
Gto	0.010	0.007	0.006	0.005	0.005	0.004	0.004	0.004	0.004	0.005	0.007
Jup	-0.054	-0.041	-0.034	-0.027	-0.020	-0.014	-0.009	-0.005	-0.005	-0.009	-0.013

Regression coefficients for the fit to APD80 in the non-failing population at each BCL.

Table S15. Regression coefficients: failing, APD3080

Parameter	BCL (ms)									
	1500	1000	900	800	700	600	500	450	400	350	300
GCaL	0.394	0.354	0.352	0.351	0.352	0.356	0.373	0.403	0.467	0.611	0.820
GKr	0.760	0.813	0.824	0.835	0.846	0.856	0.861	0.853	0.826	0.709	0.311
GKs	-0.042	-0.045	-0.042	-0.039	-0.036	-0.031	-0.027	-0.027	-0.036	-0.069	-0.103
GK1	0.247	0.239	0.238	0.236	0.233	0.229	0.223	0.220	0.214	0.201	0.185
GNaCa	0.166	0.117	0.099	0.081	0.062	0.042	0.019	0.005	-0.009	-0.023	-0.054
Gto	0.173	0.152	0.145	0.137	0.127	0.116	0.105	0.101	0.101	0.109	0.117
Jup	-0.203	-0.158	-0.139	-0.118	-0.093	-0.063	-0.027	-0.003	0.028	0.077	0.138

Regression coefficients for the fit to APD3080 in the non-failing population at each BCL.

Table S16. Regression coefficients: failing, CaTD80

Parameter	BCL (ms)									
	1500	1000	900	800	700	600	500	450	400	350	300
GCaL	-0.134	-0.213	-0.251	-0.289	-0.329	-0.373	-0.420	-0.457	-0.492	-0.541	-0.578
GKr	-0.211	-0.255	-0.254	-0.252	-0.246	-0.235	-0.222	-0.201	-0.177	-0.096	0.023
GKs	-0.022	-0.030	-0.030	-0.030	-0.030	-0.029	-0.027	-0.023	-0.018	-0.003	0.002
GK1	-0.001	-0.001	-0.002	-0.001	-0.001	-0.001	0.001	0.002	0.008	0.029	0.039
GNaCa	0.090	0.134	0.149	0.168	0.188	0.210	0.232	0.244	0.252	0.250	0.250
Gto	-0.018	-0.025	-0.027	-0.028	-0.029	-0.030	-0.031	-0.030	-0.030	-0.028	-0.023
Jup	-0.959	-0.910	-0.885	-0.855	-0.816	-0.767	-0.709	-0.678	-0.651	-0.624	-0.607

Regression coefficients for the fit to CaTD80 in the non-failing population at each BCL.

Table S17. Regression coefficients: failing, CaTD3080

Parameter	BCL (ms)									
	1500	1000	900	800	700	600	500	450	400	350	300
GCaL	0.410	0.306	0.212	0.103	-0.017	-0.144	-0.273	-0.358	-0.436	-0.522	-0.580
GKr	-0.670	-0.729	-0.687	-0.613	-0.511	-0.390	-0.263	-0.186	-0.115	-0.004	0.117
GKs	-0.073	-0.091	-0.085	-0.076	-0.063	-0.047	-0.029	-0.017	-0.005	0.015	0.016
GK1	0.000	0.002	0.002	0.003	0.003	0.003	0.004	0.005	0.011	0.029	0.036
GNaCa	0.000	0.081	0.116	0.150	0.180	0.204	0.222	0.230	0.232	0.228	0.228
Gto	0.028	0.013	0.005	-0.002	-0.008	-0.014	-0.019	-0.020	-0.021	-0.019	-0.020
Jup	0.534	0.144	0.003	-0.139	-0.270	-0.380	-0.463	-0.503	-0.527	-0.527	-0.518

Regression coefficients for the fit to CaTD3080 in the non-failing population at each BCL.

Table S18. Regression coefficients: failing, CaTmax

Parameter	BCL (ms)									
	1500	1000	900	800	700	600	500	450	400	350	300
GCaL	0.792	0.790	0.785	0.780	0.777	0.776	0.781	0.784	0.795	0.810	0.840
GKr	-0.098	-0.092	-0.089	-0.087	-0.083	-0.074	-0.056	-0.044	-0.037	-0.082	-0.155
GKs	-0.014	-0.015	-0.015	-0.015	-0.015	-0.014	-0.012	-0.011	-0.011	-0.020	-0.019
GK1	0.002	0.002	0.002	0.002	0.003	0.003	0.002	0.001	-0.005	-0.028	-0.040
GNaCa	-0.476	-0.433	-0.417	-0.404	-0.392	-0.382	-0.375	-0.370	-0.366	-0.354	-0.350
Gto	0.065	0.058	0.055	0.051	0.048	0.045	0.043	0.041	0.039	0.037	0.033
Jup	0.260	0.270	0.280	0.287	0.291	0.292	0.285	0.290	0.284	0.261	0.225

Regression coefficients for the fit to CaTmax in the non-failing population at each BCL.

Table S19. Regression coefficients: failing, AP-CaT delay

Parameter	BCL (ms)									
	1500	1000	900	800	700	600	500	450	400	350	300
GCaL	-0.696	-0.688	-0.691	-0.693	-0.698	-0.707	-0.722	-0.731	-0.741	-0.745	-0.743
GKr	0.116	0.098	0.093	0.089	0.083	0.076	0.067	0.064	0.071	0.115	0.180
GKs	0.013	0.013	0.013	0.013	0.012	0.012	0.011	0.011	0.012	0.018	0.018
GK1	-0.006	-0.006	-0.006	-0.006	-0.007	-0.007	-0.006	-0.005	-0.001	0.013	0.026
GNaCa	0.324	0.294	0.287	0.282	0.278	0.276	0.273	0.271	0.267	0.256	0.251
Gto	-0.025	-0.027	-0.027	-0.027	-0.027	-0.027	-0.027	-0.027	-0.027	-0.025	-0.025
Jup	-0.586	-0.611	-0.611	-0.609	-0.604	-0.593	-0.569	-0.549	-0.524	-0.489	-0.461

Regression coefficients for the fit to AP-CaT delay in the non-failing population at each BCL.

R^2 coefficients

Table S20. Non-failing R^2 values

Biomarker	BCL (ms)									
	1500	1000	900	800	700	600	500	450	400	350	300
APD80	0.988	0.985	0.985	0.985	0.985	0.986	0.988	0.989	0.990	0.990	0.989
APD3080	0.940	0.942	0.949	0.957	0.966	0.973	0.979	0.982	0.984	0.987	0.985
CaTD80	0.983	0.987	0.987	0.988	0.989	0.990	0.991	0.992	0.992	0.992	0.993
CaTD3080	0.803	0.864	0.865	0.860	0.852	0.840	0.831	0.842	0.861	0.890	0.920
CaTmax	0.943	0.957	0.960	0.959	0.955	0.949	0.948	0.945	0.944	0.939	0.926
AP-CaT delay	0.957	0.953	0.953	0.953	0.954	0.953	0.940	0.908	0.857	0.808	0.814

R^2 values for each fit to the non-failing population.

Table S21. Failing R^2 values

Biomarker	BCL (ms)									
	1500	1000	900	800	700	600	500	450	400	350	300
APD80	0.981	0.981	0.981	0.981	0.981	0.981	0.982	0.983	0.983	0.981	0.979
APD3080	0.901	0.914	0.918	0.923	0.929	0.937	0.947	0.954	0.961	0.946	0.885
CaTD80	0.988	0.954	0.932	0.903	0.868	0.825	0.780	0.766	0.759	0.753	0.761
CaTD3080	0.909	0.663	0.539	0.435	0.371	0.361	0.407	0.468	0.532	0.601	0.658
CaTmax	0.933	0.894	0.877	0.861	0.848	0.838	0.834	0.836	0.847	0.858	0.880
AP-CaT delay	0.943	0.940	0.938	0.936	0.933	0.928	0.919	0.910	0.897	0.872	0.835

R^2 values for each fit to the failing population.

References

1. Sobie E (2009) Parameter sensitivity analysis in electrophysiological models using multivariable regression. Biophys J 96: 1264–1274.

2. Sarkar A, Sobie E (2010) Regression analysis for constraining free parameters in electrophysiological models of cardiac cells. PLOS Comput Biol 6: e1000914.

3. Gwathmey G, Copelas L, MacKinnon R, Schoen F, Feldman M, et al. (1987) Abnormal intracellular calcium handling in myocardium from patients with end-stage heart failure. Circ Res 61: 70–76.

4. Beuckelmann D, Nabauer M, Erdmann E (1992) Intracellular calcium handling in isolated ventricular myocytes from patients with terminal heart failure. Circulation 85: 1046–1055.

5. Beuckelmann D, Nabauer M, Erdmann E (1993) Alterations of K+ currents in isolated human ventricular myocytes from patients with terminal heart failure. Circ Res 73: 379–385.

6. Vahl C, Bonz A, Timek T, Hagl S (1994) Intracellular calcium transient of working human myocardium of seven patients transplanted for congestive heart failure. Circ Res 74: 952–958.

7. Sipido K, Stankovica T, Flameng W, Vanhaecke J, Verdonck F (1998) Frequency dependence of Ca2+ release from the sarcoplasmic reticulum in human ventricular myocytes from end-stage heart failure. Cardiovasc Res 37: 478–488.

8. Kubo H, Marguiles K, Piacentino III V, Gaughan J, Houser S (2001) Patients with end-stage congestive heart failure treated with β-adrenergic receptor antagonists have improved ventricular myocyte calcium regulatory protein abundance. Circulation 104: 1012–1018.

9. Piacentino III V, Weber C, Chen X, Weisser-Thomas J, Marguiles K, et al. (2003) Cellular basis of abnormal calcium transients of failing human ventricular myocytes. Circ Res 92: 651–658.

10. Li G, Lau C, Leung T, Nattel S (2004) Ionic current abnormalities associated with prolonged action potentials in cardiomyocytes from diseased human right ventricles. Heart Rhythm 4: 460–468.

11. Glukhov A, Fedorov V, Lou Q, Ravikumar V, Kalish P, et al. (2010) Transmural dispersion of repolarization in failing and nonfailing human ventricle. Circ Res 106: 1–11.

12. Lou Q, Fedorov V, Glukhov A, Moazami N, Fast V, et al. (2011) Transmural heterogeneity and remodeling of ventricular excitation-contraction coupling in human heart failure. Circulation 123: 1881–1890.

13. Glukhov A, Fedorov V, Lou Q, Janks D, Ravikumar V, et al. (2012) Conduction remodeling in human end-stage nonischemic left ventricular cardiomyopathy. Circulation 125: 1835–1847.
